# Bridging PCR: An Efficient and Reliable Scheme Implemented for Genome-Walking

**DOI:** 10.3390/cimb45010033

**Published:** 2023-01-05

**Authors:** Zhiyu Lin, Cheng Wei, Jinfeng Pei, Haixing Li

**Affiliations:** 1 School of Chemistry and Chemical Engineering, Nanchang University, Nanchang 330031, China; 2 Sino-German Joint Research Institute, Nanchang University, Nanchang 330047, China; 3 State Key Laboratory of Food Science and Technology, Nanchang University, Nanchang 330047, China

**Keywords:** bridging PCR, genome-walking, bridging primer, walker primer, inverted repeat, intra-strand annealing

## Abstract

The efficacy of the available genome-walking methods is restricted by low specificity, high background, or composite operations. We herein conceived bridging PCR, an efficient genome-walking approach. Three primers with random sequences, inner walker primer (IWP), bridging primer (BP), and outer walker primer (OWP), are involved in bridging PCR. The BP is fabricated by splicing OWP to the 5′-end of IWP’s 5′-part. A bridging PCR set is constituted by three rounds of amplification reactions, sequentially performed by IWP, BP plus OWP, and OWP, respectively pairing with three nested sequence-specific primers (SSP). A non-target product arising from IWP alone undergoes end-lengthening mediated by BP. This modified non-target product is a preferentially formed hairpin between the lengthened ends, instead of binding with shorter OWP. Meanwhile, a non-target product, triggered by SSP alone or SSP plus IWP, is removed by nested SSP. As a result, only the target DNA is accumulated. The efficacy of bridging PCR was validated by walking the *gadA/R* genes of *Levilactobacillus brevis* CD0817 and the *hyg* gene of rice.

## 1. Introduction

Genome-walking is referred to a strategy used to retrieve unknown flanking sequences starting from a known DNA [1,2]. Genome-walking has been frequently exploited in biology-related research works, such as isolating regulatory sequence of a functional gene, cloning full-length of an open reading frame, mining new gene-resources according to a conserved region, and identifying insertion sites of a transgene [3,4,5,6,7].

Numerous genome-walking approaches have been devised to fulfill the above tasks. The genome library-based method is the earliest DNA-walking protocol. This method is rather time-consuming and low in efficiency due to requiring the construction and screening of a genomic DNA library, and therefore has been deprecated [8,9].

Later, PCR-based genome-walking methods were brought forward. Based on the involved rationales, these methods could be classified into inverse PCR [10,11], digestion and ligation-dependent PCR [12,13], and randomly primed PCR [14,15,16]. Prior to PCR amplification, the former two types require digestion of genomic DNA with a suitable endonuclease, followed by self-circularization of the digested product or ligation to a random DNA [10,12,13]. Comparatively, randomly primed PCR is an easy-to-use method because it is free of any extra operation before PCR amplification [17,18].

A randomly primed PCR relies on a set of unilaterally nested amplification reactions. The principle in devising this PCR is to reduce amplification efficiency of non-target DNA as far as possible, on the premise of ensuring that of target DNA. After two to three consecutive nested PCRs, the target DNA becomes the major product [3,19]. Thermal asymmetric interlaced PCR [14], wristwatch PCR [2], and partially overlapping primer-based PCR [8], are representatives of randomly primed strategy. However, a non-target background arising from walker primer alone cannot be efficiently eliminated from these methods [9,18].

We herein report bridging PCR, a convenient and reliable genome-walking strategy. The associated rationale is that a DNA fragment, terminated by a 40 nt or longer inverted repeat (IR), is a preferentially formed hairpin via intra-strand annealing, instead of being amplified by a shorter primer corresponding to the outmost half of the IR. As a result, only the target DNA is efficiently boosted in bridging PCR. We confirmed the feasibility of bridging PCR by obtaining the unknown segments flanking *Levilactobacillus brevis* CD0817 decarboxylase genes *gadA/R* [20] and rice hygromycin gene *hyg* [8].

## 2. Materials and Methods

### 2.1. Genomic DNA

*L. brevis* CD0817 was cultured according to the previously reported method [21,22]. The genomic DNA of this strain was extracted using the Bacterial Genomic DNA Isolation Kit (Tiangen Biotech Co., Ltd., Beijing, China) following the supplier’s guidance. The genomic DNA of rice was kindly given by the lab of Dr. Xiaojue Peng at Nanchang University (Nanchang, China).

### 2.2. Primer Design

A walker primer set three primers (IWP, OWP, and BP) were designed as follows. IWP (50 nt) and OWP (25 nt) are random oligonucleotides heterologous to each other, with Tm values of 70–75 °C and 60–65 °C, respectively. BP was made by attaching OWP to the 5′ end of IWP’s 5′ part (25 nt) (Table 1). In this work, we devised three walker primer sets so as to perform three parallel bridging PCRs in one walking. The three IWPs have identical 5′ regions (25 nt) but completely different 3′ regions (25 nt). The homology of the 5′ regions makes the OWP universal to the corresponding IWPs (Table 1), while the heterologous 3′ regions endow IWPs with personalized annealing patterns in the low-stringency cycle. Therefore, the three sets were actually constituted by five primers, that is, three IWPs, one BP, and one OWP. Three nested SSPs having a Tm of 60–65 °C were selected from each gene. All the primers and primer pairs were prevented from forming severe hairpin or dimer.

### 2.3. Bridging PCR Procedure

Each walking comprised of three sets of bridging PCRs. Each bridging PCR set consisted of three rounds (primary, secondary, and tertiary) of amplification reactions. Genomic DNA was employed as template in primary PCR conducted by SSP1 and IWP. The 50-μL primary reaction system included 1 × LA PCR buffer II, 1 μL genomic DNA (microbe, 10–100 ng; or rice, 100–1000 ng), 0.4 mM each dNTP, 0.2 μM each primer, and 2.5 U of LA Taq polymerase (TaKaRa, Beijing, China).

An aliquot (1 μL) of primary product was directly employed as template of secondary reaction driven by SSP2 and OWP plus BP. The 50-μL secondary reaction mixture was composed of 1 × LA PCR buffer II, 1 μL primary product, 0.4 mM each dNTP, 0.2 μM OWP, 0.2 μM SSP2, 8 nM BP, and 2.5 U LA Taq polymerase. It is worthy to be emphasized that the working concentration of BP is much lower than that of the other both primers.

Likewise, 1 μL secondary product was used as template of tertiary PCR performed by SSP3 and OWP. The 50-μL tertiary reaction mixture contained 1 × LA PCR buffer II, 1 μL secondary product, 0.4 mM each dNTP, 0.2 μM each primer, and 2.5 U LA Taq polymerase.

The primary bridging PCR consisted of three annealing phases: phase 1, five high-stringency (65 °C) cycles; phase 2, one low-stringency (25 °C) cycle; and phase 3, 30 high-stringency cycles. The secondary and tertiary PCRs performed 25–45 and 15–30 high-stringency cycles, respectively. The detailed thermal cycling parameters for bridging PCR are summarized in Table 2.

### 2.4. DNA Manipulation and Sequencing

The PCR products underwent electrophoretic separation on 1.5% agarose gel. The clear DNA band(s) in a secondary or tertiary PCR was recovered using the MiniBEST Agarose Gel DNA Extraction Kit Ver.4.0 (TaKaRa, Beijing, China) according to the guidance. The recovered products were sent to the Sangon Biotech Co., Ltd. (Shanghai, China) for direct sequencing.

## 3. Results

### 3.1. Rationale and Process of Bridging PCR

**Figure 1:**
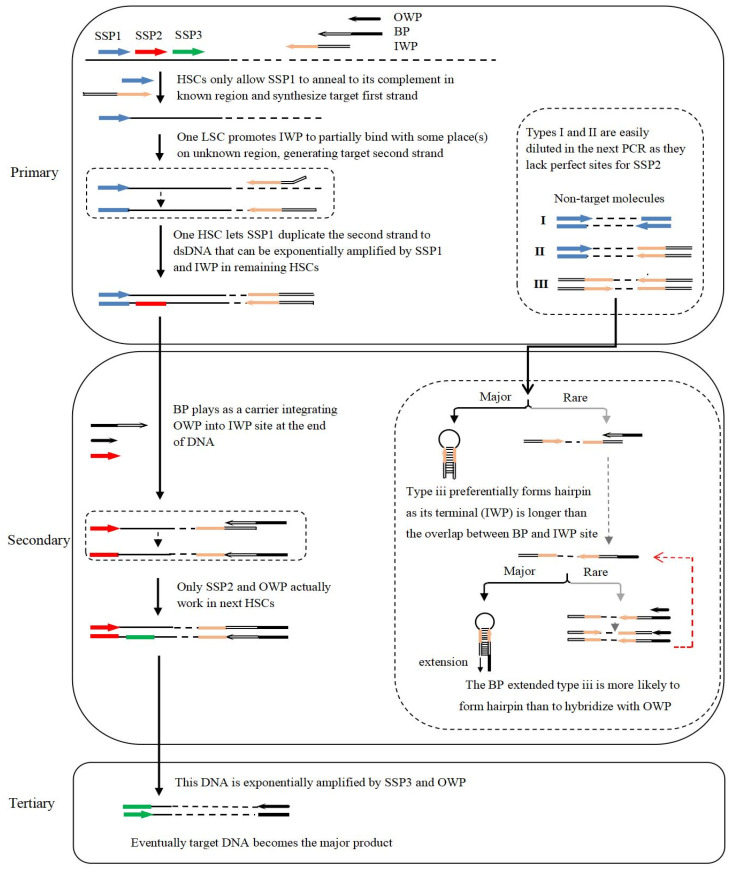
Schematic diagram of bridging PCR. ISP: inner walker primer; BP: bridging primer; OWP: outer walker primer; and SSP: sequence-specific primer. Thin solid lines: known sequences; thin dotted lines: unknown sequences; color thick arrows: primers; color thick lines: primers complements; ssDNA: single-stranded DNA; dsDNA: double-stranded DNA; HSC: high-stringency cycle; LSC: low-stringency cycle.

The rationale and experimental process of bridging PCR are displayed in Figure 1. In primary PCR the initial five high-stringency cycles, only sequence-specific primer 1 (SSP1) binds with its complement in the known region and synthesizes first strand of interest, theoretically obtaining a five-fold increase in target template. The subsequent one low-stringency cycle promotes inner walker primer (IWP) to partially anneal to some place(s) on the first strand, generating the second strand. The second strand is duplicated by SSP1 into double-stranded DNA (dsDNA) in the subsequent one high-stringency cycle. Any non-target single-stranded (ssDNA), however, cannot be converted into dsDNA given that it lacks an authentic site for the primers. The target dsDNA is exponentially enriched in the remaining high-stringency cycles, while the non-target background is ruled out. Still, three types of non-target molecules will be presumably produced, due to the complexity of genome, the use of random IWP, and the execution of low-stringency cycle. Type i is produced by SSP1 alone; type ii is produced by SSP1 plus IWP; and type iii is produced by IWP alone.

Three primers, out walker primer (OWP), bridging primer (BP), and SSP2, are included in the secondary PCR using primary PCR product as template. OWP and SSP2 are used with a normal concentration (0.2 μM), while the concentration of BP is as low as 8 nM. Clearly, only OWP and SSP2 can actually perform amplification reaction, while BP acts only as a vehicle to integrate OWP into the end(s) of DNA terminated by IWP. The current method is thus called bridging PCR. Once OWP is incorporated into target DNA, it will be exponentially amplified by SSP2 and OWP. The aforementioned type i non-target DNA resulting from SSP1 alone is entirely removed, as it lacks a perfect binding site for the primers. After OWP is attached to the IWP site, type ii non-target produced by SSP1 and IWP can only be linearly amplified by OWP. Type iii non-target generated by IWP alone can be connected with OWP at both ends. The resultant DNA is formed hairpin between the lengthened terminal IR, instead of hybridizing with OWP shorter than the IR. As a result, the amplification of type iii non-target is also inhibited (Figure 1). In summary, the secondary bridging PCR exclusively allows the target DNA to be enriched.

Tertiary PCR is implemented by SSP3 and OWP, adopting secondary amplification product as template. The experimental process and the mechanism of removing non-target DNA are similar to secondary PCR (Figure 1), which will not be described here. Finally, the target DNA becomes the major product, meanwhile any non-desired DNA is removed.

### 3.2. Effects of Dilution of Amplification Product on Next PCR

**Figure 2:**
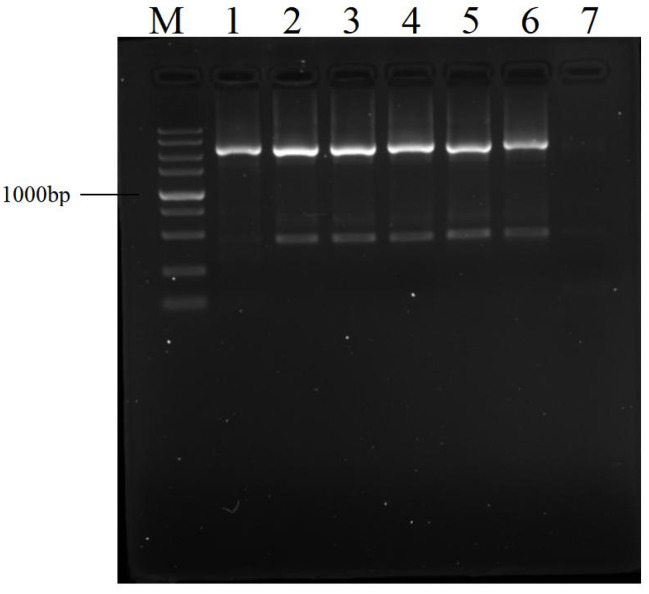
Effects of dilution of primary amplification product on secondary PCR. The amplified target was *gadR*. The primary PCR was performed as described in Table 2. Lane 1: primary PCR product; lanes 2–6: secondary PCR products using 0, 10, 100, 1000, and 10,000-fold diluted primary products as templates, respectively; lane 7: secondary PCR result without primary product; and lane M: DNA 5000 Marker (5000, 3000, 2000, 1500, 1000, 750, 500, 250, and 100 bp).

To determine whether an amplification product needs to be diluted before the next PCR, *gadR* (Table 1) was selected to undergo bridging PCR. The primary PCR was implemented according to the thermal cycling conditions listed in Table 2. The 10-fold serial dilutions of primary product were used as templates of secondary PCRs with 30 cycles. As shown in Figure 2, 0- to 1/10,000-fold dilutions give almost identical amplification outcomes, although DNA band becomes weaker with the dilution rate exceeding 1000 folds. As expected, no outcome was produced in the case of no primary product.

### 3.3. Effects of PCR Cycle Number

**Figure 3:**
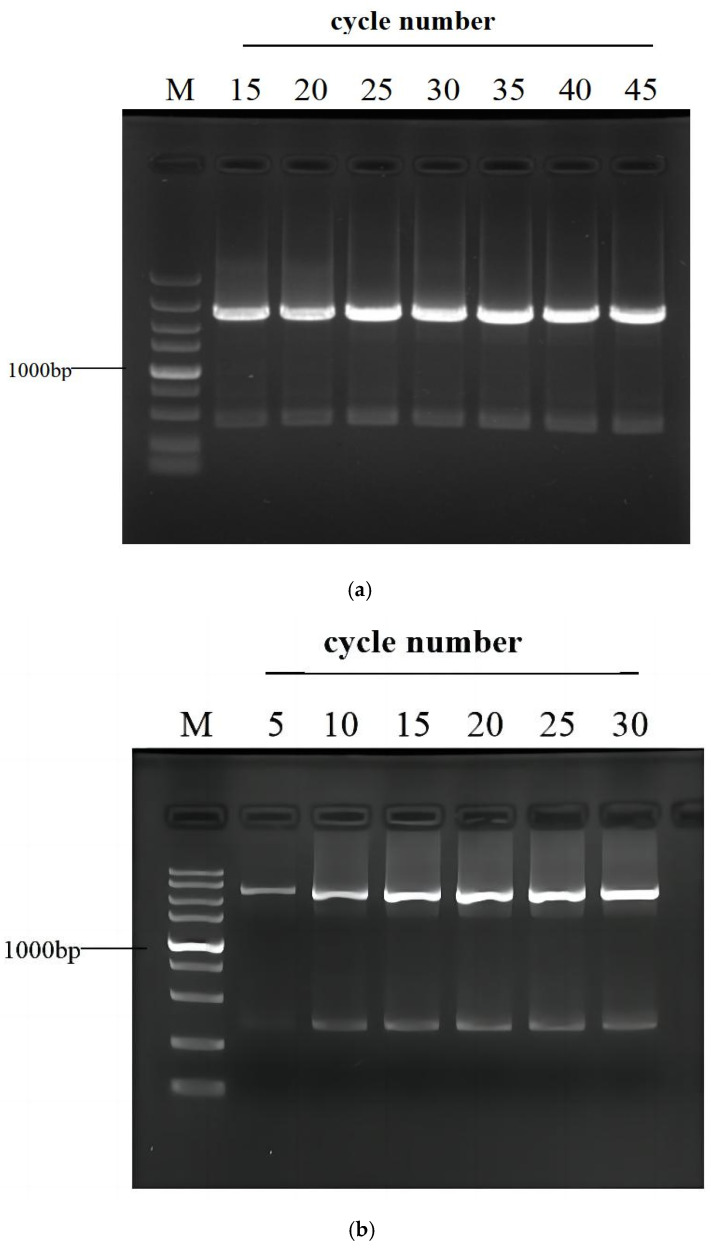
Effects of cycle number on (**a**) Secondary or (**b**) tertiary PCR. The primary amplification of target *gadR* was performed as shown in Table 2. The numbers on the top of the electropherograms represent PCR cycle numbers. Lane M: DNA 5000 Marker (5000, 3000, 2000, 1500, 1000, 750, 500, 250, and 100 bp).

Secondary or tertiary PCRs respectively run various numbers of cycles so as to determine the appropriate cycle number. For this aim, *gadR* was adopted as the amplified target. The primary PCR was executed according to Table 2. The secondary PCRs, directly using 1 μL of primary product as templates, were conducted for 15, 20, 25, 30, 35, 40, and 45 cycles, respectively. The tertiary PCRs, directly using 1 μL of 30-cycle secondary product as templates, were conducted for 5, 10, 15, 20, 25, and 30 cycles, respectively. The results demonstrated that all the secondary PCRs generate the same amplification pattern, but a PCR of 25 or more cycles facilitates a sufficient amplification. Likewise, a tertiary PCR with at least 15 cycles is suggested (Figure 3).

### 3.4. Validation of Bridging PCR

**Figure 4:**
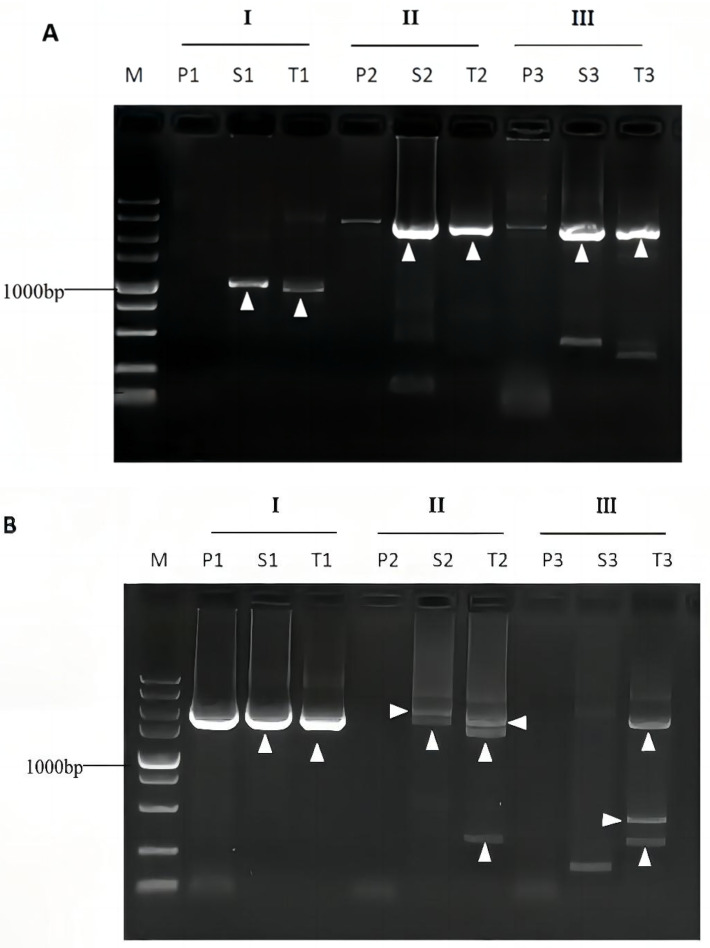

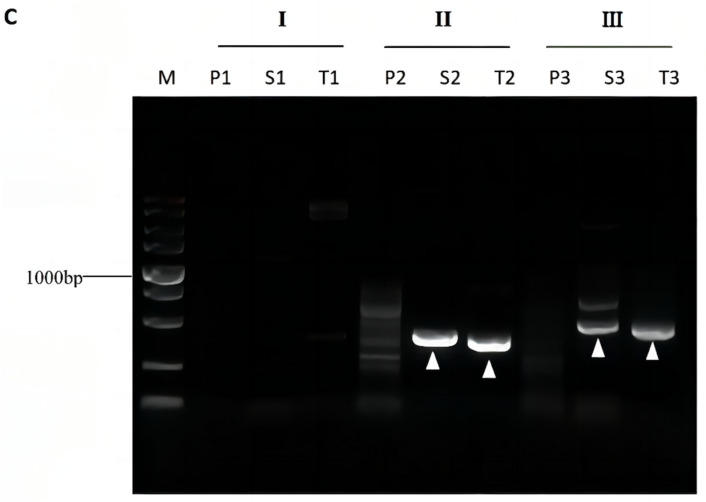
Genome-walking for *gadA* (**A**) and *gadR* (**B**) of *L. brevis* CD0817, and *hyg* (**C**) of rice. I, II, and III denote the three parallel sets of bridging PCRs in each walking experiment. Lanes P: primary PCR; S: secondary PCR; T: Tertiary PCR; and M: DNA5000 Marker (5000, 3000, 2000, 1500, 1000, 750, 500, 250, and 100 bp). The white arrowheads indicate the clear major bands.

We exploited this method to access unknown sequences flanking *Levilactobacillus brevis* CD0817 glutamate decarboxylase genes *gadA/R* and the rice hygromycin gene *hyg*. A SSP set three nested SSPs were selected from each gene. In this work, three walker primer sets were designed and individually paired with a SSP set to perform three parallel bridging PCRs for each walking (Table 1). The 3′ parts (25 nt) of the three IWPs are heterologous to each other so that each has an individualized annealing pattern on an unknown flank. The PCR reaction systems and experimental operations are as depicted in the section of Materials and Methods. As shown in Figure 4, all the positive secondary or tertiary PCRs generated only one to two discrete major DNA band(s), with a negligible background. All these major bands were recovered using the MiniBEST Agarose Gel DNA Extraction Kit Ver.4.0. The recovered products were directly sequenced. The sequencing data demonstrated that all the bands were the wanted products, because one end of a product overlaps the corresponding known region.

## 4. Discussion

In a randomly primed PCR, three types of non-target products are commonly generated: type i, arising from SSP alone; type ii, arising from the combination of SSP and walker primer; and type iii, arising from walker primer alone [14,19]. Types i and ii can be easily omitted by nested SSP(s). Type iii non-target DNA, the major origin of background in randomly primed PCR, cannot be routinely removed with nested SSP(s), challenging the amplification specificity of this method. As a result, type iii non-target product has seriously perplexed the randomly primed PCR-based genome-walking approaches [2,14,18].

In the current bridging scheme, a suppression PCR was introduced to overcome type iii non-target fragment. In the case that a DNA is surrounded by a long IR (~40 bp) while the primer only corresponds to the outmost half of this IR, the DNA is prone to form a hairpin mediated by the IR. As a result, the suppression effect on PCR amplification occurs [23,24]. In the proposed bridging strategy, the BP serves only as a medium attaching OWP to the 5′-end of IWP, and thereafter inhibits the amplification of type iii non-target DNA by OWP (Figure 2). Meanwhile, the non-target amplification resulting from BP is also negligible because of its low concentration and 25 nt shorter than the terminal IR comprising IWP and OWP.

The bridging PCR has showed a satisfactory amplification specificity. The existing randomly primed PCRs, such as partially overlapping primer-based PCR [8], wristwatch PCR [2], or fusion primer PCR [18], cannot efficiently overcome non-target product accumulation, because they are based on the partial overlapping between walker primers. Apparently, a non-target DNA can gain the same amplification efficiency as the target one, as long as the walker primer is incorporated into the former walker primer site [18,19]. The thermal asymmetric interlaced PCR has to use one low-stringency cycle in every three cycles, meaning non-specific amplification is not negligible [14,15]. In contrast, the inhibition of non-target amplification occurs in each thermal cycle of bridging PCR, as shown in Figure 1. The experimental results confirmed the high specificity of this method, which was reflected by the negligible electrophoretic background and the recovered amplicons that can be directly sequenced. This merit of bridging PCR abolishes the thereafter DNA cloning step that is popular in the traditional PCRs [2,14].

The bridging PCR has a high success rate. This method adopts one low-stringency (25 °C) cycle in primary PCR. At such a low temperature, IWP should identify at least one locus on an unknown flank suitable for its partial annealing. Once this partial annealing occurs, the walking is expected to succeed [2,8,9]. Furthermore, parallel experiments provide an extra success guaranteeing mechanism for bridging PCR. We suspect that at least one bridging PCR would yield positive results if more than one is simultaneously performed. With regards to the success probability, bridging PCR should be comparable to the partially overlapping primer-based PCR [8], thermal asymmetric interlaced PCR [14], and wristwatch PCR [2]. Another benefit from parallel experiments is that a long target amplicon may be obtained in a single walking experiment. Different IWPs should have individualized annealing patterns because their 25 nt of 3′ regions are heterologous to each other. Therefore, it cannot be excluded that some primer(s) may anneal on unknown region to a place far from known DNA [2,18].

The multi-band phenomenon common in routine randomly primed PCRs is substantially improved in bridging PCR. In each round of PCR of a routine method, at least one low-stringency (25–40 °C) cycle has to be conducted. At such a low temperature, the primers will hybridize to many places on DNA of interest, resulting in multi-band phenomenon [2,14,25]. In contrast, all the amplification cycles in secondary or tertiary bridging PCR are performed under high-stringent conditions, thus overcomes internal annealing of the primers to target DNA (Figure 1). This merit of bridging PCR improves multi-band phenomenon prevailing in the existing randomly primed PCRs. The validation experiments verified this, because all the positive secondary/tertiary PCRs released just one to two (only one in most cases) target product (Figure 4). However, the existing methods generated more DNA bands [2,8].

The bridging PCR is a convenient and universal way of genome-walking, requiring neither digestion or ligation operation, nor dilution of the PCR product prior to next amplification (Figure 3). In addition, a walker primer set used in the current approach are entirely random and versatile to any genome. What users need to do is just select nested SSPs according to a known DNA. The prevailing randomly primed PCRs generally have to carry out three rounds of nested amplifications so as to obtain a convincing result [14,15,17]. For bridging PCR, the tertiary amplification is generally not required, as the secondary PCR suffices to give a positive outcome (Figure 4).

In summary, an efficient and reliable bridging PCR approach has been set up for genome-walking. The proposed method should be a promising alternative to the traditional genome-walking schemes.

## Figures and Tables

**Table 1:** Primers used in this study.

	Primary PCR	Secondary PCR	Tertiary PCR
Walker primer set	IWP1: GTCGTAGTCATGTATCGTCCTAGTCATCTGCTTGTTCGTCAGTCAGCGTC	BP: *CAGTCAGTCTCAGCTAGTCAGTGTC*GTCGTAGTCATGTATCGTCCTAGTC OWP: *CAGTCAGTCTCAGCTAGTCAGTGTC*	OWP: *CAGTCAGTCTCAGCTAGTCAGTGTC*
	IWP2: GTCGTAGTCATGTATCGTCCTAGTCTCAGTCAGTCAGTTGCAGTCAGTCT		
	IWP3: GTCGTAGTCATGTATCGTCCTAGTCATCCAGAACAGTCGATTGGTTCAGC		
*gadA* SSP set	SSP1: TCCAAGAATCATCCGCAATCGTCA	SSP2: TGGTAACATCGTCACGGTTCTTTGG	SSP3: TAGCCTTGTACCCATCTTTACCGAA
*gadR* SSP set	SSP1: TCCTTCGTTCTTGATTCCATACCCT	SSP2: CCATTTCCATAGGTTGCTCCAAGG	SSP3: GGATACTGGCTAAAATGAATTAACTCGGATAA
*hyg* SSP set	SSP1: ACGGCAATTTCGATGATGCAGCTTG	SSP2: GGGACTGTCGGGCGTACACAA	SSP3: CTGGACCGATGGCTGTGTAGAAG

Note: The three IWPs own the identical 5′ regions (25 nt, underlined), and the heterologous 3′ regions. The BP was generated by attaching OWP (italic) to the 5′-end of IWP’s underlined 5′-region. The three IWPs individually pair with a SSP1 to perform three parallel primary PCRs. The three secondary PCRs, individually using primary PCR product as templates, are conducted by a SSP2 and the universal BP and OWP. The three tertiary PCRs, individually using secondary PCR product as templates, are conducted by a SSP3 and the OWP. IWP: inner walker primer; BP: bridging primer; OWP: outer walker primer; SSP: sequence-specific primer; *gad*: glutamic acid decarboxylase gene; and *hyg*: hygromycin gene.

**Table 2:** Thermal cycling parameters of bridging PCR.

Round of PCR	Thermal Cycling	Cycle Number
Primary	95 °C, 1 min	
	95 °C 20 s, 65 °C 30 s, 72 °C 2 min	5
	95 °C 20 s, 25 °C 30 s, 72 °C 2 min	1
	95 °C 20 s, 65 °C 30 s, 72 °C 2 min	30
	72 °C 5 min	
Secondary	95 °C, 1 min	
	95 °C 20 s, 65 °C 30 s, 72 °C 1.5 min	25–45
	72 °C 5 min	
Tertiary	95 °C, 1 min	
	95 °C 20 s, 65 °C 30 s, 72 °C 1.5 min	15–30
	72 °C 5 min	

## Data Availability

Not applicable.
